# Experimental Human Challenge Defines Distinct Pneumococcal Kinetic Profiles and Mucosal Responses between Colonized and Non-Colonized Adults

**DOI:** 10.1128/mBio.02020-20

**Published:** 2021-01-12

**Authors:** Elissavet Nikolaou, Simon P. Jochems, Elena Mitsi, Sherin Pojar, Annie Blizard, Jesus Reiné, Carla Solórzano, Edessa Negera, Beatriz Carniel, Alessandra Soares-Schanoski, Victoria Connor, Hugh Adler, Seher R. Zaidi, Caz Hales, Helen Hill, Angie Hyder-Wright, Stephen B. Gordon, Jamie Rylance, Daniela M. Ferreira

**Affiliations:** a Department of Clinical Sciences, Liverpool School of Tropical Medicine, Liverpool, United Kingdom; b Department of Parasitology, Leiden University Medical Center, Leiden, Netherlands; c Bacteriology Laboratory, Butantan Institute, São Paulo, Brazil; d Department of Respiratory Medicine, Royal Liverpool University Hospital, Liverpool, United Kingdom; e Malawi-Liverpool Wellcome Trust Clinical Research Programme, Blantyre, Malawi; Carnegie Mellon University

**Keywords:** *Streptococcus pneumoniae*, colonization, saliva, nasal lining fluid, cytokines, neutrophil acquisition, host-pathogens interactions, controlled human infection

## Abstract

Colonization of the upper respiratory tract with Streptococcus pneumoniae is the precursor of pneumococcal pneumonia and invasive disease. Following exposure, however, it is unclear which human immune mechanisms determine whether a pathogen will colonize. We used a human challenge model to investigate host-pathogen interactions in the first hours and days following intranasal exposure to Streptococcus pneumoniae. Using a novel home sampling method, we measured early immune responses and bacterial density dynamics in the nose and saliva after volunteers were experimentally exposed to pneumococcus. Here, we show that nasal colonization can take up to 24 h to become established. Also, the following two distinct bacterial clearance profiles were associated with protection: nasal clearers with immediate clearance of bacteria in the nose by the activity of pre-existent mucosal neutrophils and saliva clearers with detectable pneumococcus in saliva at 1 h post challenge and delayed clearance mediated by an inflammatory response and increased neutrophil activity 24 h post bacterial encounter. This study describes, for the first time, how colonization with a bacterium is established in humans, signifying that the correlates of protection against pneumococcal colonization, which can be used to inform design and testing of novel vaccine candidates, could be valid for subsets of protected individuals.

## Abstract (precis)

Occurrence of lower respiratory tract infections requires prior colonization of the upper respiratory tract with a pathogen. Most bacterial infection and colonization studies have been performed in murine and *in vitro* models due to the current invasive sampling methodology of the upper respiratory tract, both of which poorly reflect the complexity of host-pathogen interactions in the human nose.

## INTRODUCTION

The human respiratory tract is a major site of contact with aerosolized bacteria. Acute respiratory tract infections are common, and pneumonia causes more than 1.3 million child deaths annually (1, 2) as well as frequent hospitalizations in at-risk groups, such as the elderly, people with chronic lung disease, and asthmatics (3).

The first stage of such infections is the successful colonization of the upper respiratory tract by the pathogen (4). Streptococcus pneumoniae, the major bacterial cause of pneumonia, inhabits the nasopharynx of 40 to 95% of young children and 10 to 25% of adults without causing disease (5–7). Colonization is usually asymptomatic in adults but can be associated with mild rhinitis symptoms in children (8). Different serotypes of pneumococcus may inhabit the nasopharynx at varying densities (9, 10). Colonization may continue for a period of weeks or months, and S. pneumoniae colonization is eliminated and reacquired many times during life (11). As pneumococcal colonization is the primary reservoir for transmission (12) and a prerequisite of invasive disease (13), its control is key to preventing disease. Importantly, which factors determine whether exposure to S. pneumoniae leads to colonization have not been completely identified in humans. Factors such as host age, immune status, virus coinfection, exposure to antibiotics, smoking, and overcrowded living conditions have all been associated with increased susceptibility to colonization (14–16).

The Experimental Human Pneumococcal Challenge (EHPC) model allows for the rapid, safe, and accurate study of bacterial encounter at the nasopharynx in humans (17). In the EHPC model, the precise dose and timing of infection are known. Individuals are inoculated with live type 6B pneumococcus, and pneumococcal colonization (detection and density) is assessed by nasal washes collected from 48 h onwards post exposure (17). After this point, approximately 40 to 50% of individuals become carriers, but the early kinetics of bacterial clearance or colonization onset have not been assessed. In this study, healthy adult volunteers were challenged with S. pneumoniae, after which they self-collected saliva and nasal lining fluid (NLF) samples for bacterial kinetics and mucosal immune monitoring in the first 48 h. We compared non-colonized and colonized subjects to identify potential correlates of protection and to define the kinetics of colonization. Our hypothesis was that mucosal innate responses would be predictive of protection against colonization, and the movement of bacteria from the nose (site of exposure) to the saliva within the first 48 h would be associated with less effective immunity and potentially with colonization in adults.

## RESULTS

### Home sampling method can be used to collect samples at defined time points.

Sixty-three volunteers aged 18 to 49 years were screened for preexisting colonization with pneumococcus, all of whom were noncolonized, and subsequently inoculated with 6B pneumococcus as previously described (17). Colonization status was defined by classical microbiology culture of S. pneumoniae serotype 6B in nasal wash samples collected at days 2, 6, 9, 14, 21, and 27 post exposure. Volunteers with negative samples at all time points were defined as culture-negative, and those with a positive sample at any time point were classified as culture-positive. Following exposure, 41 volunteers remained culture-negative (41/63, 65.1%) and 22 became culture-positive (see Table S1 in the supplemental material). Colonization status was confirmed by S. pneumoniae 6A/B capsule-specific and *lytA*-specific quantitative PCR (qPCR) in nasal wash to ensure that we did not miss low-density colonizers (18). Only one culture-negative volunteer showed a low positive signal (threshold cycle [*C_T_*] = 37) at day 6 post S. pneumoniae exposure.

10.1128/mBio.02020-20.5 TABLE S1 Volunteer demographic data. Sixty-three volunteers aged 18 to 49 years, 41 culture-negatives and 22 culture-positives. Download Table S1, DOCX file, 0.01 MB. Copyright © 2021 Nikolaou et al. 2021 Nikolaou et al. This content is distributed under the terms of the Creative Commons Attribution 4.0 International license.

**FIG 1:**
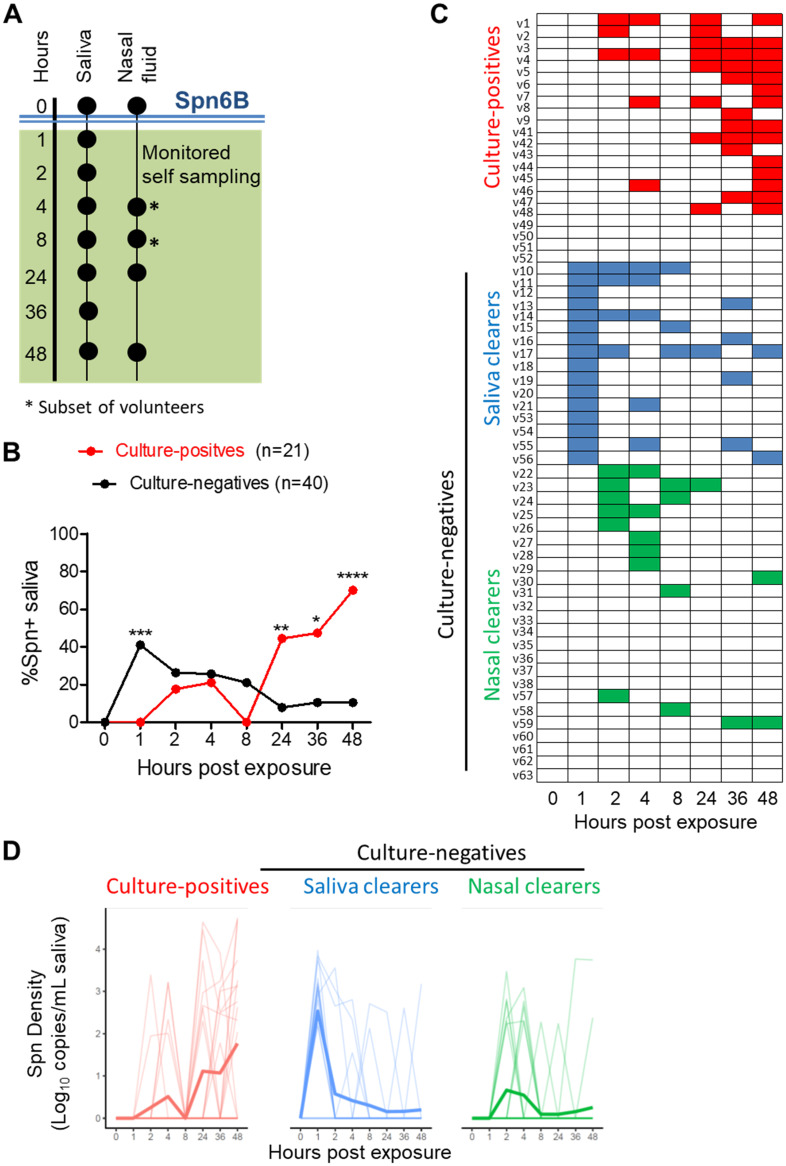
Kinetics of early pneumococcal detection in saliva. (A) Schematic representation of sample collection design. Saliva and NLF samples were collected before exposure, *T* = 0 h (baseline), in clinic. Volunteers were asked to self-collect saliva at 1, 2, 4, 8, 24, 36, and 48 h and NLF at 24 and 48 h post exposure. *, A subgroup of 33 individuals also collected NLF at 4 and 8 h post exposure to assess very early nasal microbiological and immunological dynamics. (B) Frequency of volunteers with detected S. pneumoniae 6B pneumococcus in saliva. Pneumococcal DNA presence in saliva was determined by S. pneumoniae (Spn) 6A/B qPCR. The number of volunteers with S. pneumoniae 6B presence (*C_T_* < 40) in each time point is expressed as a percentage (%) of the total number of volunteers for culture-positive and culture-negative groups in red and black, respectively. Statistical significance based on Fisher’s exact test. *T* = 1 h, ***, *P* = 0.0005; *T* = 24 h, **, *P* = 0.002; *T* = 36 h, *, *P* = 0.011; and *T* = 48 h, ****, *P* < 0.0001. (C) Heatmap showing individual saliva profiles. Presence of pneumococcal DNA detected from raw and/or culture-enriched extraction is depicted. Three distinct saliva profiles were defined. Culture-positive, volunteers who were identified to be experimentally colonized with pneumococcus at day 2 or later using classical microbiology (red, *n* = 21). Culture-negatives were divided into two groups as follows: saliva clearers, volunteers with detectable S. pneumoniae in saliva at 1 h after exposure (blue, *n* = 16) and nasal clearers, volunteers without detectable S. pneumoniae in saliva at 1 h after exposure (green, *n* = 24). (D) Density levels of pneumococcal 6A/B PCR in saliva (culture-positives, *n* = 21; saliva clearers, *n* = 16; and nasal clearers, *n* = 24). S. pneumoniae 6B density was expressed as DNA copies per volume (ml) of saliva. Only DNA from extractions without culture enrichment were included, as enrichment modifies density values (see also Fig. S2D). All samples with undetectable S. pneumoniae were set to 0 CFU/ml. Data were log transformed after adding 1 to all values to allow transformation of 0 values. Individual volunteers and the mean of log-transformed values are shown.

Saliva and NLF samples were obtained before exposure (time = 0 h, baseline) (Fig. 1A). Volunteers collected their own saliva into preprepared tubes at 1, 2, 4, 8, 24, 36, and 48 h, and NLF (by nasosorption strip) (19) at 24 and 48 h. A subset of 33 volunteers self-collected in addition NLF samples at 4 and 8 h to assess very early nasal dynamics (see Fig. S1 in the supplemental material). To monitor compliance, volunteers were instructed to record sample collection times using their mobile phones and send pictures of the collected sample to the research team at the time of sample collection (see Fig. S2A in the supplemental material). Samples from two volunteers were excluded (3.2%) due to incorrect storage of samples, as at least half of their samples were stored at room temperature (Fig. S2B). Therefore, data from 61 volunteers were evaluated in this study as follows: 40 culture-negative and 21 culture-positive volunteers (Table S1). This high rate of compliance (61/63, 96.8%) indicates the successful application of this self-sampling method.

10.1128/mBio.02020-20.1 FIG S1 Flow chart depicting the samples processing for pneumococcal detection in the study. Sixty-three healthy young adults age 18 to 49 years were recruited. Two volunteers were excluded from the initial cohort due to incorrect sample storage as detected by temperature monitoring, thus 61 volunteers were used in this analysis. Colonization status was defined by classical microbiology culture (blood agar plates with gentamycin) of S. pneumoniae serotype 6B in nasal wash samples collected before (day −5) and at days 2, 6, 9,14, 21, and 27 postexposure. Twenty-one volunteers were classified as culture-positives, as they had at least one positive sample at any time point during the study, whereas 40 volunteers had negative samples at all time points and were classified as culture-negatives. Saliva and NLF samples were obtained before exposure (time = 0 h, baseline, day of exposure) from all volunteers. Volunteers self-collected their own saliva at 1, 2, 4, 8, 24, 36, and 48 h and NLF (by nasosorption strip) at 24 and 48 h postexposure. Pneumococcal presence was detected in both saliva and NLF samples by extracting pneumococcal genomic DNA from raw material. In addition, for saliva samples, DNA was extracted from culture-enriched (CE) (blood agar plate with gentamicin) saliva samples. *, A subset of 33 volunteers self-collected in addition NLF samples at 4 and 8 h (66 samples) post exposure; these numbers are not included in this diagram. **, Samples from 7 volunteers (5 culture-positives, 40 samples; and 2 culture-negatives, 16 samples) were used for optimization experiments and were not included for culture-enriched extraction. From two of these seven volunteers (1 culture-positive, 3 samples; and 1 culture-negative, 3 samples), no bacterial DNA was extracted from NLF. Download FIG S1, DOCX file, 0.1 MB. Copyright © 2021 Nikolaou et al. 2021 Nikolaou et al. This content is distributed under the terms of the Creative Commons Attribution 4.0 International license.

10.1128/mBio.02020-20.2 FIG S2 Home sampling of saliva and nasal lining fluid for S. pneumoniae detection. (A) Compliance monitoring for one example volunteer (v23). Temperature loggers were used to measure sample temperature, and recordings were taken every 20 s to ensure sample stability. Volunteers sent pictures of collected samples directly after sample collection to demonstrate that they were taken at the correct time. Planned times relative to experimental S. pneumoniae inoculation are shown and compared with the times of pictures for one representative volunteer. (B) Pie chart of compliance rates for all 63 volunteers. The number of corrected stored samples was counted per volunteer, expressed as percentage (%) of the total number of samples, and used to categorize compliance status. Compliance status was then expressed as percent compliance to the total number of volunteers. Excellent, 100% of samples stored at fridge/freezer; very good, 80 to 90% of samples stored at fridge/freezer; good, 70 to 80% of samples stored at fridge/freezer; and poor, ≤50% of samples stored at fridge/freezer from time of collection. (C) Venn diagram of S. pneumoniae detection using qPCR in culture-enriched saliva versus raw extracted saliva. Saliva samples from 7 volunteers (v4, v5, v7, v8, v9, v21, v38 with 17 positive S. pneumoniae timepoints) were excluded, as no culture-enrichment step was performed for these volunteers, and DNA from all material was already extracted before they were plated. (D) Comparison of S. pneumoniae 6B density from raw extracted and culture-enriched saliva samples. Open circles are samples detected by both methods (*n* = 27). S. pneumoniae density in samples detected by culture-enriched extraction was statistically significantly higher than that detected by raw DNA extraction (****, *P* < 0.0001, paired *t* test). Black circles are samples detected only by raw DNA extraction (*n* = 32), and grey circles are samples detected only by culture-enriched DNA extraction (*n* = 24). Download FIG S2, DOCX file, 0.2 MB. Copyright © 2021 Nikolaou et al. 2021 Nikolaou et al. This content is distributed under the terms of the Creative Commons Attribution 4.0 International license.

### Raw and culture-enriched DNA extractions in saliva were complementary for pneumococcal detection in saliva.

To detect S. pneumoniae presence, pneumococcal genomic DNA was extracted from both raw and culture-enriched (blood agar plate with gentamicin) saliva samples, as the latter might lead to increased pneumococcal detection in saliva (20, 21). In total, 83 time points with S. pneumoniae present were detected using an S. pneumoniae 6A/B capsule-specific qPCR (22): 32 only in raw samples, 24 only in culture-enriched samples, and 27 in both (Fig. S2C). Consequently, samples with a positive result using either method were included in further analyses. As expected, S. pneumoniae density from culture-enriched samples was significantly higher (lower *C_T_* values) than that from raw samples (Fig. S2D).

### Two distinct profiles of bacterial clearance kinetics associated with protection against colonization.

To investigate the kinetics of S. pneumoniae clearance and establishment, we assessed the presence of pneumococcal DNA in saliva in the first 48 h after exposure (Fig. 1A). At 1 h post challenge, S. pneumoniae DNA was detected in the saliva of 16/40 (40%) culture-negative volunteers and 0/21 (0%) culture-positive volunteers (Fig. 1B and C; ***, *P* = 0.0005, Fisher Exact test). Thus, a subset of culture-negative volunteers exhibited rapid movement of S. pneumoniae to the saliva following exposure, which could suggest that two distinct profiles of protection against colonization exist. We named these two groups “nasal clearers” (individuals with no detectable S. pneumoniae DNA in 1 h post exposure saliva samples) and “saliva clearers” (individuals with detectable S. pneumoniae DNA in 1 h post exposure saliva samples). Saliva clearers showed a peak of S. pneumoniae density (DNA copies/ml saliva) at 1 h after exposure, followed by a gradual decrease of S. pneumoniae density after 1 h. Nasal clearers showed similar levels of S. pneumoniae density between 2 and 12 h (Fig. 1D).

### Establishment of pneumococcal colonization takes up to 24 h.

Among the culture-positive volunteers, S. pneumoniae DNA was not detected in any 1 h saliva samples (Fig. 1B and C). At 2, 4, and 8 h, detection of S. pneumoniae DNA was not different from that of those who become culture-negative. At 24 h, however, S. pneumoniae DNA was detected in saliva of 8/21 (38.1%) culture-positive volunteers but only 2/40 (5%) of culture-negative volunteers (Fig. 1B and C; **, *P* = 0.002). Moreover, the proportion of culture-positives with detectable S. pneumoniae DNA in the saliva continued to increase to 14/21 (76.2%) at 48 h (Fig. 1B; ****, *P* < 0.0001 compared to culture-negatives). At the same time, the mean density of S. pneumoniae DNA started increasing in culture-positive volunteers from 24 h onwards (Fig. 1D). This suggests that bacterial amplification only starts occurring after this period and that S. pneumoniae colonization following experimental exposure is a gradual colonization process that takes at least 24 h.

### Bacterial DNA detection in the nose post exposure.

**FIG 2:**
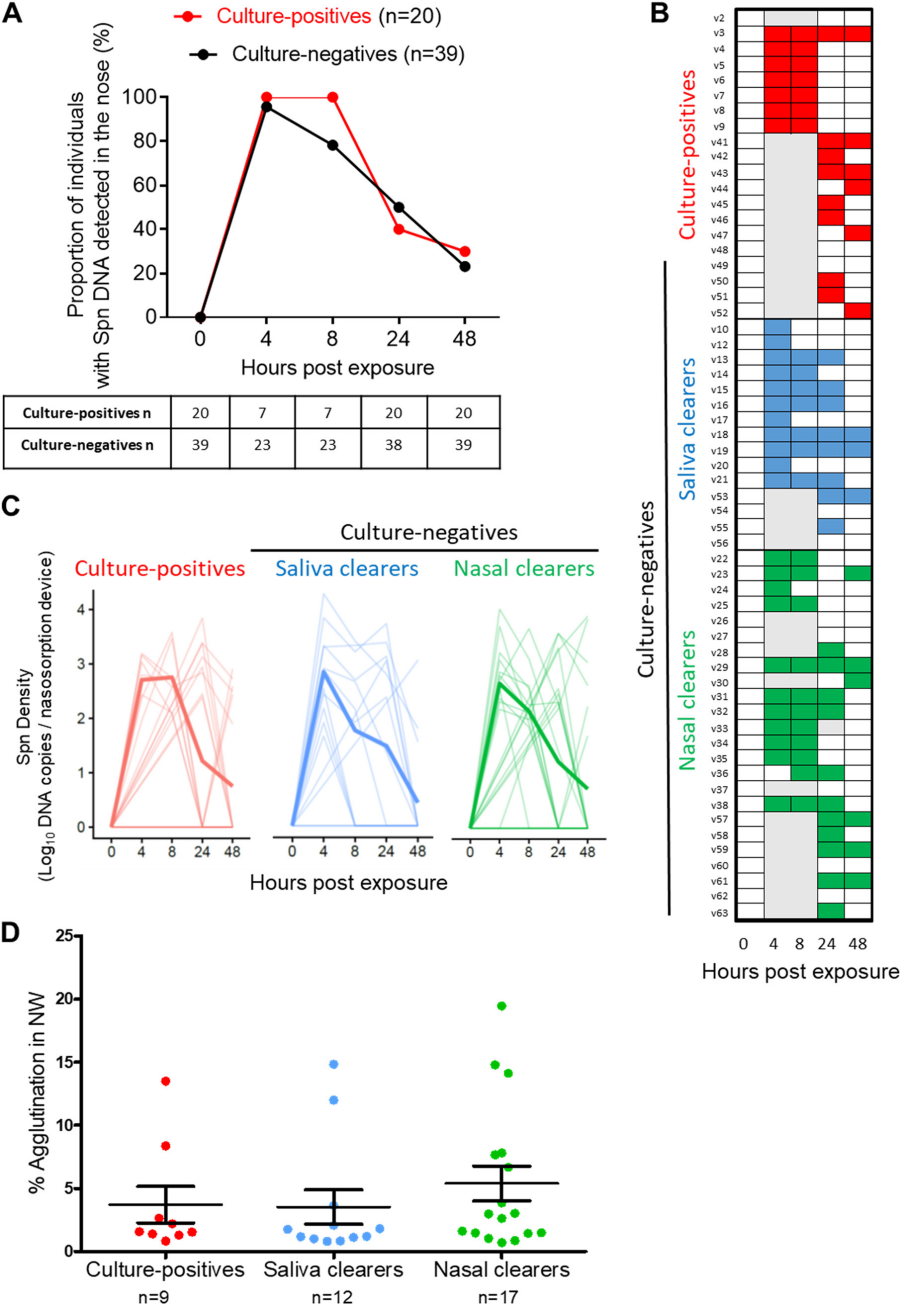
Kinetics of pneumococcal detection in nasal fluid following exposure. (A) Frequency of S. pneumoniae (Spn) DNA detection in nasal fluid after challenge, stratified by final colonization status (determined by conventional bacterial culture methods). NLF samples were collected before (*T* = 0) pneumococcal exposure and at 4, 8, 24, and 48 h post exposure. The number of volunteers with S. pneumoniae 6B presence in each time point is expressed as a percentage (%) of the total number of volunteers analyzed for culture-positives (red) and culture-negatives (black). (B) Individual-level DNA detection results, grouped by culture-positives (red, *n* = 20) and culture-negatives (*n* = 39). The latter group is subdivided into saliva clearers (blue, *n* = 15) and nasal clearers (green, *n* = 24). Samples not taken are highlighted in gray. (C) Density levels of pneumococcal 6A/B PCR in NLF, expressed as DNA copies per nasosorption device. If no S. pneumoniae was detected, the density was set as 0 CFU/ml. Data was log transformed after adding 1 to all values to allow transforming 0 values. Individual volunteers and the mean of log-transformed values are shown. (D) Levels of agglutination capacity in nasal wash at baseline in initial cohort. Nine culture-positive, 12 saliva clearer, and 17 nasal clearer volunteers were included in analysis. Each dot represents one volunteer. Mean ± standard error of the mean (SEM) is shown for each of the three groups.

We then evaluated the presence of pneumococcal DNA in the nasal lining fluid during the first 48 h post exposure by qPCR and stratified this by colonization status determined by conventional bacterial culture at later time points. At 4 and 8 h, S. pneumoniae DNA was detected in almost all NLF samples (Fig. 2A and B). At 24 and 48 h post exposure, S. pneumoniae DNA was detected in nasal lining fluid from 27/58 (47%) and 15/59 (25%) total volunteers, respectively. When stratified, there was no difference in DNA detection rates in the first 2 days between those who were subsequently colonized and those who were not (Fig. 2A to C). An absence of detectable bacterial DNA at 24 h suggests bacterial clearance or migration posteriorly in the nasopharynx or attachment and internalization at the epithelium (Fig. 2A and B).

### Pneumococcal agglutination was not associated with early pneumococcal profiles.

**FIG 3:**
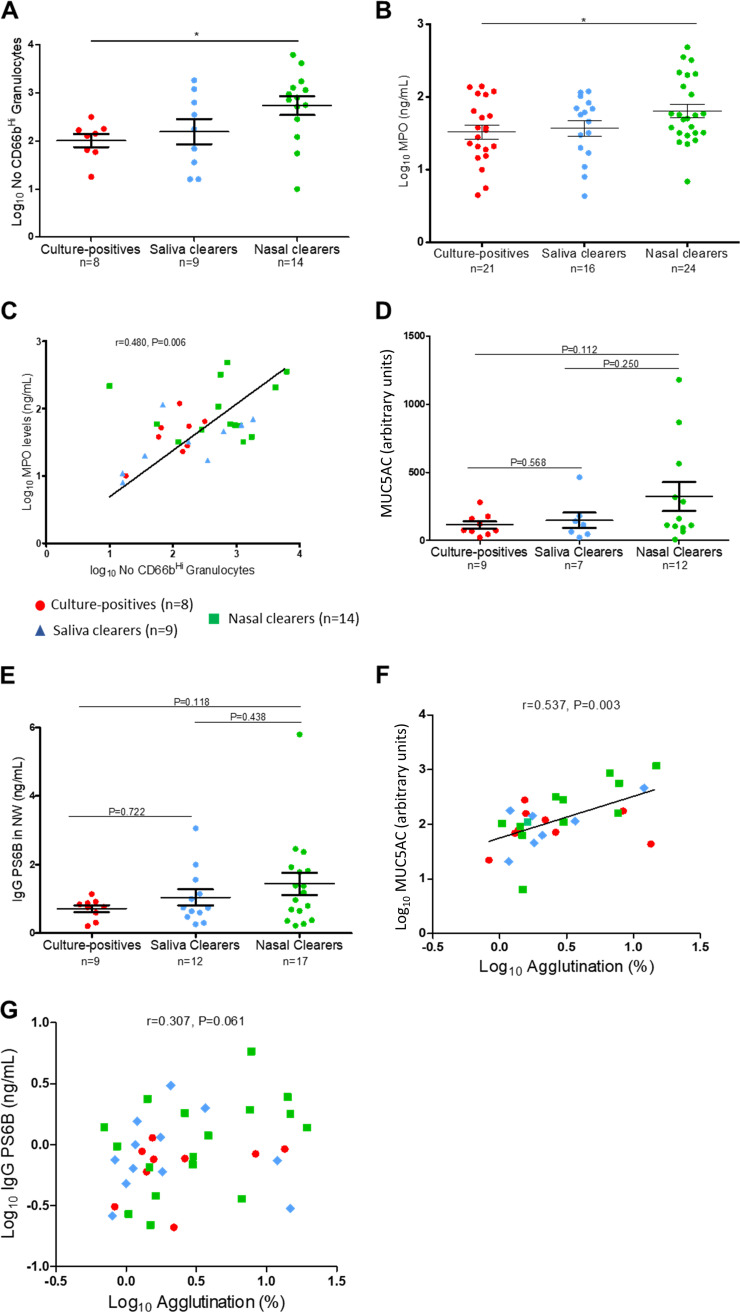
Association of baseline mucosal immune factors with early pneumococcal colonization profiles. (A) Neutrophil number in nasal scrapes prior to pneumococcal exposure. Abundance of CD66b-high cells (activated granulocytes) at baseline were measured by flow cytometry. Eight culture-positives, 9 saliva clearers, and 14 nasal clearers were assessed (*, *P* = 0.018, unpaired *t* test). There is no statistically significant difference of the CD66b^Hi^ neutrophil counts between nasal and saliva clearers (*P* = 0.107, unpaired *t* test). Data were log transformed, and individual volunteers and mean ± SEM are represented. (B) MPO levels in nasal wash prior to pneumococcal exposure—both cohorts. MPO levels at baseline were measured by ELISA. Twenty-one culture-positives, 16 saliva clearers, and 24 nasal clearers were assessed. MPO levels in nasal wash were significantly increased in nasal clearers compared to those in culture-positives (*, *P* = 0.034, unpaired *t* test). Data were log transformed, and individual volunteers and mean ± SEM are represented. (C) Correlation between MPO levels in nasal wash and number of activated granulocytes (CD66b^Hi^) in nasal scrapes prior to S. pneumoniae challenge for paired samples. Eight culture-positives, 9 saliva clearers, and 14 nasal clearers were assessed (Pearson test, *r* = 0.480; **, *P* = 0.006). Data were log transformed. Culture-positives, red circles; saliva clearers, blue rectangular; and nasal clearers, green squares. (D) MUC5AC in nasal wash at baseline. Each dot represents a volunteer, and mean ± SEM are shown. No statistical significance was detected within the groups (unpaired *t* test, culture-positives versus saliva clearers, *P* = 0.568; culture-positives versus nasal clearers, *P* = 0.112; saliva clearers versus nasal clearers, *P* = 0.250). (E) Levels of S. pneumoniae 6B polysaccharide-specific IgG (PS6B) antibodies in nasal wash at baseline. Each dot represents a volunteer, and mean ± SEM are shown. No statistical significance was detected within the groups (Mann-Whitney test, culture-positives versus saliva clearers, *P* = 0.722; culture-positives versus nasal clearers, *P* = 0.118; saliva clearers versus nasal clearers, *P* = 0.438). (F) Agglutination capacity (%) versus mucin (MUC5AC) in nasal wash at baseline. Culture-positives, red circles (*n* = 9); saliva clearers, blue rectangular (*n* = 7); and nasal clearers, green squares (*n* = 12). Data were log transformed, and Spearman rho and *P* values are depicted in addition to linear regression (black line). (G) Agglutination capacity (%) versus S. pneumoniae 6B polysaccharide-specific IgG (PS6B) antibody levels in nasal wash at baseline. Culture-positives, red circles (*n* = 9); saliva clearers, blue rectangular (*n* = 12); and nasal clearers, green squares (*n* = 17). Data were log transformed, and Spearman rho and *P* values are indicated.

Agglutination of pneumococcus by polysaccharide 6B (PS6B)-specific IgG antibodies has been previously shown to protect against 6B pneumococcus colonization in the context of pneumococcal conjugate vaccination (23). Also, mucus is known to be able to trap pathogens (24) and to bind pneumococcus through carbohydrate motifs (25). We hypothesized that the saliva clearer group had a superior agglutination capacity, leading to rapid detection of S. pneumoniae DNA in saliva and, therefore, measured agglutination capacity in baseline nasal wash samples (Fig. 2D). We also associated agglutination capacity with concentrations of mucin 5AC (MUC5AC) and S. pneumoniae 6B polysaccharide-specific IgG antibodies (PS6B) in baseline nasal wash samples (Fig. 3D and E). Baseline nasal wash agglutination capacity was correlated significantly with high levels of MUC5AC (Fig. 3F) but not with PS6B-specific IgG levels (Fig. 3G). However, agglutination capacity and levels of MUC5AC and PS6B-specific IgG were not significantly different between the three groups in nasal wash at baseline.

### Neutrophil activity contributes to protection against establishment of colonization in nasal clearers.

Neutrophils are abundantly present in the adult human nose even in the absence of pneumococcal colonization or symptoms (19). Neutrophils are activated by bacterial encounter, releasing myeloperoxidase (MPO) during degranulation (26, 27). To investigate if neutrophil activity at the time of bacterial encounter can protect against establishment of pneumococcal colonization, the abundance of neutrophils and MPO levels were measured at baseline. Nasal immune and epithelial cells were measured from nasal curettes from a subset of 40 volunteers (see Fig. S3A in the supplemental material) (19, 28). The absolute number of activated neutrophils were identified by measuring CD66b^Hi^ granulocyte levels, a marker for neutrophil activation (29). We also analyzed total number of granulocytes and the expression levels of CD66b^Hi^ within the granulocyte population (Fig. S3A).

10.1128/mBio.02020-20.3 FIG S3 Nasal immune and epithelial cells measured from nasal curettes at baseline. (A) Gating strategy for one representative volunteer. (B) Numbers of granulocytes at baseline. *, *P* = 0.038, unpaired *t* test. (C) Mean fluorescent intensity (MFI) of CD66b on granulocytes. *, *P* = 0.047, Mann-Whitney. (D) Numbers of B cells at baseline. (E) Numbers of T cells at baseline. (F) Numbers of epithelial cells at baseline. (G) Numbers of monocytes at baseline. Each dot represents a volunteer. Data were log transformed after adding 1 to all values to allow transforming 0 values and are represented as mean ± SEM. Download FIG S3, DOCX file, 0.4 MB. Copyright © 2021 Nikolaou et al. 2021 Nikolaou et al. This content is distributed under the terms of the Creative Commons Attribution 4.0 International license.

At baseline, nasal clearers had increased numbers of activated (CD66b^Hi^) granulocytes (median, 759; interquartile range [IQR]: 343 to 1,248) compared to those of culture-positives (median, 134; IQR: 64 to 171) from nasal curettes (Fig. 3A). Moreover, total numbers of granulocytes were also increased (median, 5,978; IQR: 3,268 to 8,678) in curettes of nasal clearers compared to those of culture-positives (median, 1,701; IQR: 589 to 2,261) (Fig. S3B). This finding was supported by increased MPO levels at baseline in nasal wash in nasal clearers compared to those of culture-positives (Fig. 3B). Moreover, the granulocytes of nasal clearers had increased expression of CD66b compared to those of saliva clearers (Fig. S3C). There was a significant correlation between numbers of CD66b^Hi^ granulocytes and MPO levels in nasal wash (Fig. 3C). Furthermore, there was no significant difference in levels of any other measured cell type (B cells, T cells, epithelial cells, and monocytes) between the three groups (Fig. S3D to G), indicating that increased levels of neutrophils at baseline are protective against pneumococcal colonization in nasal clearers. Thus, high levels of neutrophils and MPO at baseline are associated with protection against pneumococcal colonization.

### A robust inflammatory response is associated with protection from colonization in saliva clearers.

**FIG 4:**
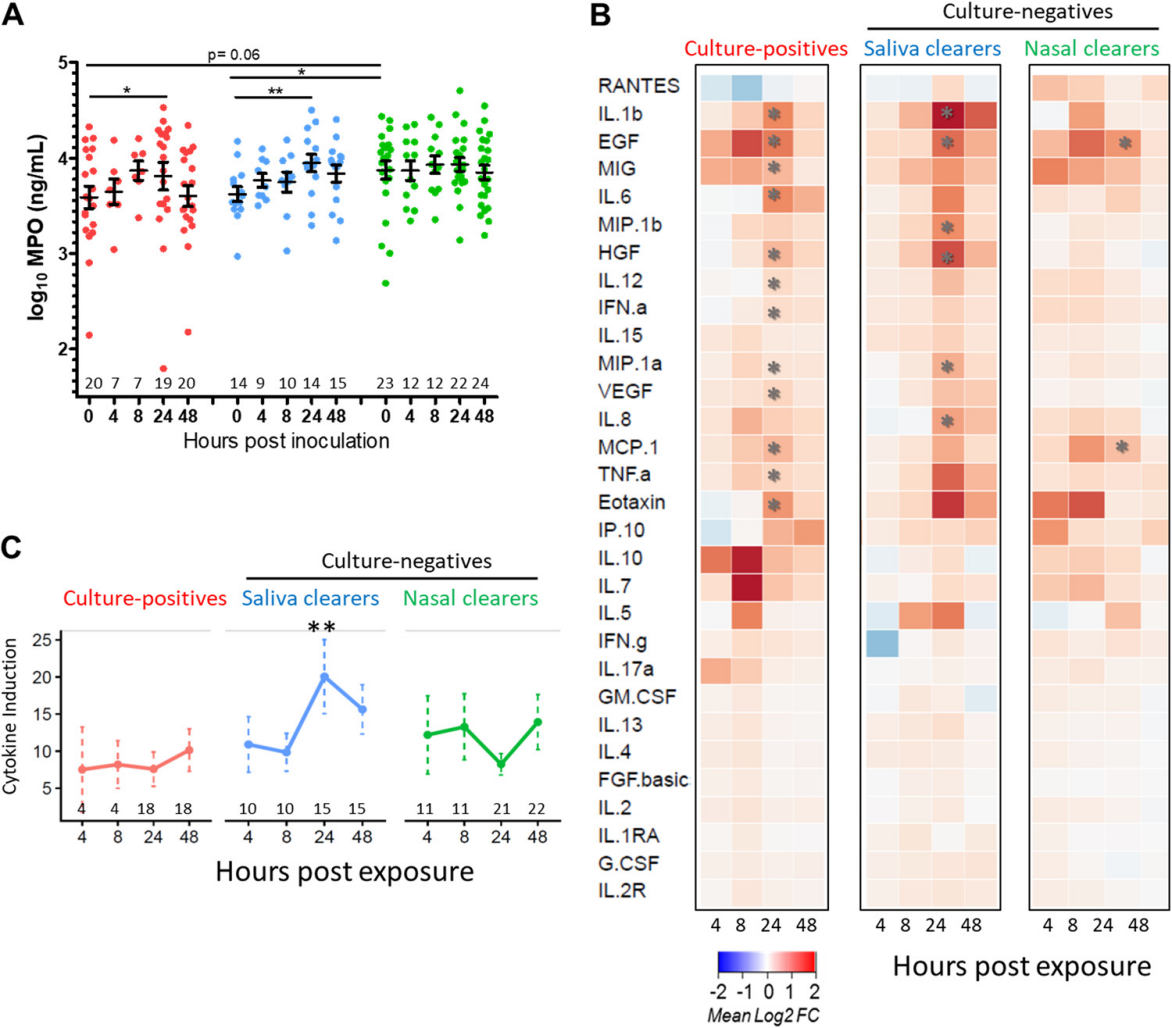
Nasal immune factors during the first 48 h post exposure. (A) MPO levels in nasal lining fluid during the first 48 h measured by ELISA. Volunteer numbers assessed are indicated above the *x* axis per time point after exposure. Data were log transformed, and individuals and mean ± SEM are depicted. In culture-positives and saliva clearers, levels of MPO rose after exposure, peaking at 24 h after pneumococcal exposure (*, *P* = 0.016 and **, *P* = 0.006, respectively; paired *t* test to baseline). MPO baseline levels are statistically significantly different between nasal clearers and saliva clearers (*, *P* = 0.046, unpaired *t* test). (B) Cytokine profile heatmap in nasal lining fluid following S. pneumoniae challenge within the three groups. Concentrations of 30 cytokines was measured by Luminex at 4, 8, 24, and 48 h after pneumococcal exposure and normalized to baseline levels for each subject. The mean of log_2_-tranformed fold changes are shown per time point for each of the three groups. *, *P* < 0.05 based on paired *t* test comparing to baseline, followed by multiple testing correction (Benjamini-Hochberg). (C) Cytokine induction score in nasal lining fluid following S. pneumoniae challenge. A total cytokine induction score was calculated by summing the Z-score normalized to fold change for each of the measured cytokines. Paired volunteer numbers to baseline are indicated per time point after exposure (same numbers in Fig. 4B). Saliva clearers showed statistically significant total induction score of cytokines at 24 h postexposure compared to those of nasal clearers and culture-positives.

To investigate whether a prompt immune response contributes to protection from colonization by pneumococcus, we longitudinally measured the abundance of 30 cytokines in nasal lining fluid using Luminex. In addition, we longitudinally measured levels of MPO in nasal lining fluid in the first 48 h post exposure (Fig. 4A). Similar to what we measured in nasal wash, MPO levels in nasal clearers were elevated at baseline in nasal lining fluid. Levels of MPO remained the same during the first 2 days in nasal clearers. In saliva clearers and culture-positives, however, levels of MPO rose after exposure and reached a significant peak at 24 h (Fig. 4A).

For the other 30 analyzed cytokines, there were no differences at baseline between the three groups (see Fig. S4A in the supplemental material). However, distinct cytokine profiles were observed over time within the three groups (Fig. 4B and C). Nasal clearer volunteers had limited cytokine induction at any time point measured during the first 48 h, with only monocyte chemoattractant protein 1 (MCP-1) and epidermal growth factor (EGF) significantly induced at 24 h post exposure, after correction for multiple testing. In contrast, saliva clearers and culture-positives showed a significant induction of multiple cytokines at 24 h post exposure. Saliva clearers showed a significant induction of 6 cytokines (interleukin-1β [IL-1β], EGF, MIP-1β/CCL4, hepatocyte growth factor [HGF], MIP-1α/CCL3, and IL-8). Culture-positives showed significant induction of 12 cytokines at this time point (Fig. 4B; IL-1β, EGF, MIG, IL-6, HGF, IL-12, alpha interferon [IFN-α], MIP-1α/CCL3, vascular endothelial growth factor [VEGF], MCP-1, tumor necrosis factor alpha [TNF-α], and eotaxin). At 48 h, no cytokines were significantly increased compared to those at baseline in any of the groups, demonstrating a transient response to S. pneumoniae exposure. A total cytokine induction score was calculated by summing the Z-score normalized fold change for each of the measured cytokines, similar to that which has previously been described for gene expression (30). Based on this cytokine induction score, saliva clearers showed an increased response upon exposure compared to that of nasal clearers and culture-positives (Fig. 4C). Cytokines that were found to be increased in any condition at 24 h strongly correlated with MPO production in nasal lining fluid at 24 h (Fig. S4B).

10.1128/mBio.02020-20.4 FIG S4 Nasal cytokine levels prior to challenge. (A) Nasal cytokines at baseline. Mean and 95% confidence intervals are shown for log-transformed cytokine concentrations (pg/ml) for culture-positives (red), nasal clearers (green), and saliva clearers (blue) in nasosorption. (B) Correlation matrix showing the association between significantly induced cytokines and MPO levels in nasosorption at 24 h in saliva clearer and culture-positive groups. Non-highly significant correlations (*P* > 0.001, Spearman test) are left blank, and rho values are shown for each cytokine pair. Color and size reflect strength of a correlation. Download FIG S4, DOCX file, 0.3 MB. Copyright © 2021 Nikolaou et al. 2021 Nikolaou et al. This content is distributed under the terms of the Creative Commons Attribution 4.0 International license.

**FIG 5:**
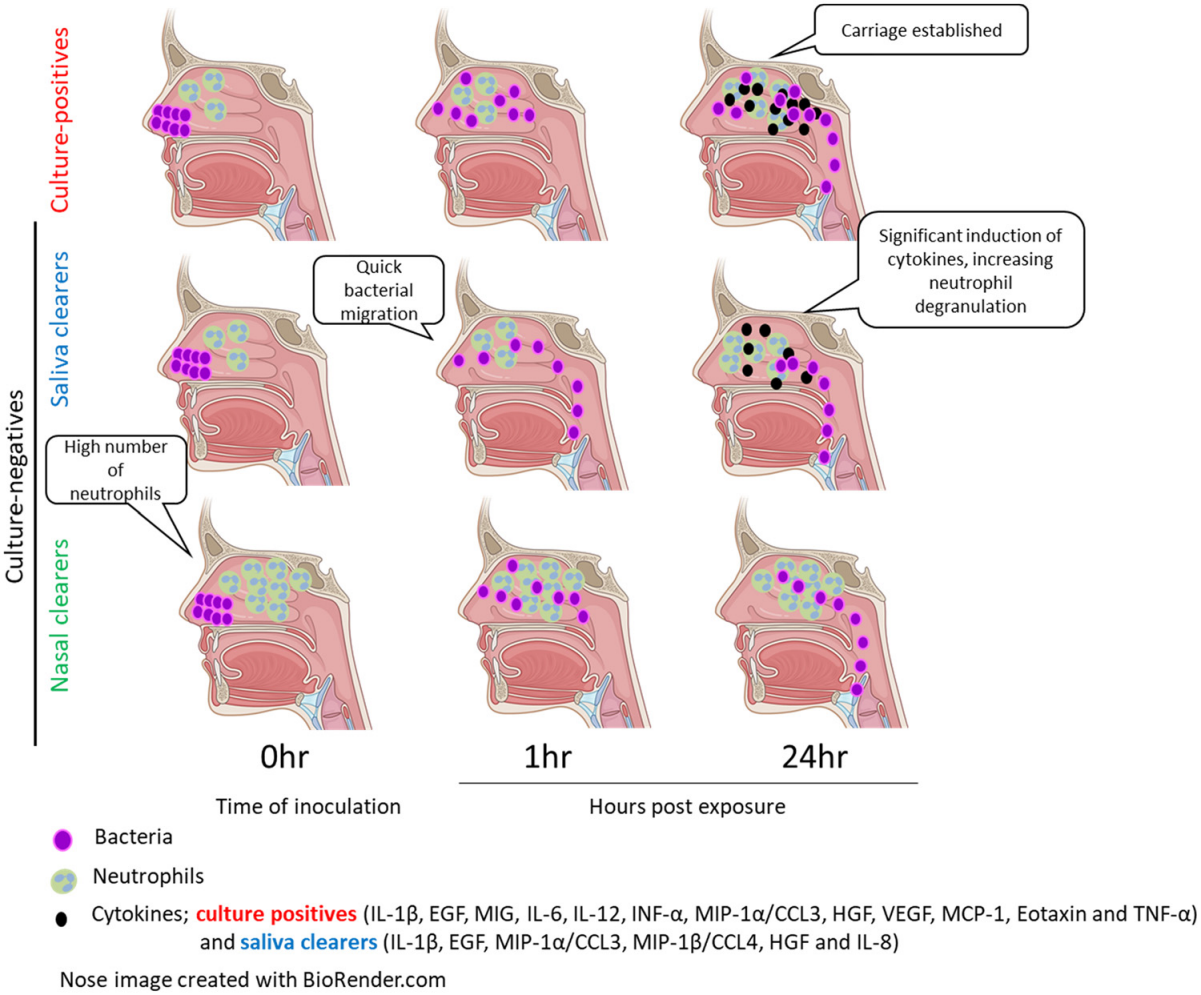
Summary model of findings. Schematic drawings of the nasopharynx are depicted for the three groups of individuals. Neutrophils, cytokines, and bacteria are depicted over time to illustrate the bacterial and immunological kinetics upon pneumococcal exposure.

Thus, nasal clearers, who had increased neutrophil levels at baseline showed limited cytokine responses upon exposure, while both saliva clearers and culture-positives showed a transient but clear response upon exposure, which was significantly higher in the saliva clearer group (Fig. 5).

## DISCUSSION

To develop effective protective interventions against disease, it is vital to understand which mechanisms play a crucial role in host-pathogen interactions by protecting against the establishment of colonization following bacterial encounter. Here, we investigated human-pneumococcus interactions in the first 48 h following controlled intranasal exposure. We tested the hypothesis that volunteers who are susceptible to colonization have a distinct profile of S. pneumoniae DNA kinetics and immune responses in the nose and saliva from those in the culture-negative group in the first hours and days after exposure.

Culture-positive volunteers showed no fast-initial clearance (appearance in saliva) during the first hour following exposure (Fig. 5). Colonization took at least 24 h to be fully established, after which increased S. pneumoniae densities could be measured in saliva. We also observed pneumococcal presence progressively diminishing in the anterior nose (exposure site) after 24 h, suggesting that migration toward the nasopharyngeal site may take place by this time point. Alternatively, the absence of pneumococcus from the anterior nose may reflect strong epithelium binding or even internalization of the bacteria. Indeed, pneumococcus was found to attach to epithelium of the inferior nasal turbinate in the EHPC model during colonization (31). One limitation to our study is that it uses an experimental challenge of adults. Therefore, findings might not be generalizable to infants or young children, as important differences in immune system and susceptibility to pneumococcus exist between adults and children (32). Moreover, it is possible that colonization dynamics during experimental challenge are different from those during natural exposure.

Nasal clearers showed a strong baseline neutrophil activation. These volunteers did not develop a significant pro-inflammatory response or had bacterial movement to the saliva within 1 h. It is possible that the high level of baseline activated nasal neutrophils prevented sensing by epithelium or other cell types, such as monocytes, as well as movement to the saliva. This indicates that neutrophils play a key role in the early control of human pneumococcal colonization. This has not been appreciated in murine models of infections, as neutrophils are abundant in the nasal lumen of humans (33) but not of naive mice (34) that show cellular influx only following bacterial challenge. In agreement with our finding, the relative prevalence of pneumococcal serotypes in causing colonization in the community associates with their relative capacities to resist neutrophil-mediated killing *in vitro* (35).

Saliva clearers (around 40% of protected individuals) were defined by an initial fast movement of the bacteria to the saliva. Although this was not associated with increased agglutination capacity, this could still be due to effective nasal mucociliary activity. Moreover, saliva clearers induced a strong proinflammatory response at 24 h post exposure. This transient response suggests that in this group, similar to culture-positives but not in nasal clearers, the inoculated bacteria is sensed, leading to an induction of immunological responses. Although culture-positives showed more significantly increased cytokines at 24 h than saliva clearers (12 versus 6), the fold change of induced cytokines was larger in the saliva clearers as reflected by the higher cytokine induction score. Moreover, cytokines IL-8 and MIP-1b were only significantly increased in the saliva clearer group. Taken together, this suggests that this group could be protected by mucociliary clearance, which is then supported by an innate response to pneumococcus.

In conclusion, we described the dynamics of establishment of colonization by S. pneumoniae in humans and observed that two different mechanisms were associated with protection. This highlights that correlates of protection against pneumococcal colonization, which can be used to inform design and testing of novel vaccine candidates, could be valid for subsets of protected individuals.

## MATERIALS AND METHODS

### Experimental design—recruitment of volunteers and ethical statements.

Volunteers were enrolled from clinical studies conducted between 2016 and 2019. Details on study design have been previously described (36). Ethical approval was given by local NHS Research and Ethics Committee (REC) (14/NW/1460, 18/NW/0481, 15/NW/0931), and the clinical trial was registered on the European Clinical Trials Database (EudraCT, 2014-004634-26 and ISRCTN22467293). All experiments conformed to the relevant regulatory standards (Human Tissue Act, 2004). Informed consent was obtained from all volunteers.

Briefly, volunteers were screened for S. pneumoniae colonization (natural carriers) and were intranasally inoculated with the S. pneumoniae 6B serotype (strain BHN418; GenBank accession number ASHP00000000.1) at 8 × 10^4^ CFU/100 μl per nostril. Colonization was assessed by classical microbiology culture in nasal washes collected at 2, 6, 9, 14, 21, and 27 days post exposure, and serotype was confirmed by latex agglutination (Statens Serum Institut, Copenhagen, Denmark). Colonization results were confirmed by S. pneumoniae 6A/B capsule-specific and *lytA*-specific qPCR on nasal wash pellet as described below. Volunteers enrolled in home sampling were not vaccinated against S. pneumoniae.

### Home sampling procedure.

Volunteers were given written instruction sheets for sample self-collection and picture taking/sending via mobile messaging application plus a sample data collection form (time planner). Volunteers were given a transport bag containing eight 10-ml saliva collection tubes and funnels (plus one spare) (Isohelix; Cell Projects Ltd, Kent, UK) with 1 ml skim milk, tryptone, glucose, and glycerin (STGG) medium with 50% glycerol for bacterial preservation and transport, 4 adsorptive matrix filter strips (Nasosorption; Hunt Developments Ltd, West Sussex, UK) for nasal fluid collection, 2 ice packs for keeping samples cooled during the day, 1 plastic box for sample storage, and a USB temperature data logger thermometer (Woodley Equipment Company Ltd, Lancashire, UK) for temperature monitoring. For nasal lining fluid collection, the matrix strip was inserted into the nostril and held against the nasal lining for 2 min and then placed in its transport tube. For saliva collection, volunteers spat up to 1 ml of saliva into the STGG tube. Both sample collection methods were demonstrated to the volunteers at baseline sample collection. Optimization experiments were performed to investigate optimal sample storage conditions by spiking saliva with different S. pneumoniae concentrations. Results indicated that S. pneumoniae density was lower in samples stored at ambient temperature; however, similar S. pneumoniae density was detected from samples stored either in a fridge or freezer. Samples taken were stored in the volunteer’s home freezer overnight and transported to the lab at their day 2 clinic visit, where they were immediately stored at −80°C until use.

### Bacterial DNA extraction from saliva samples.

Bacterial genomic DNA was extracted from raw and culture-enriched saliva samples. On the day of the extraction, saliva samples were thawed for 30 min at room temperature and vigorously vortexed for 20 s. Two hundred microliters of raw saliva was used for DNA extraction. In addition, for the culture enrichment step, 10 μl of raw saliva was diluted with 90 μl of saline and cultured on Columbia blood agar supplemented with 5% horse blood (PB0122A; Oxoid/Thermo Scientific) and 80 μl gentamicin 1 mg/ml (G1264-250mg; Sigma-Aldrich Co. Ltd). Plates were incubated overnight at 37°C and 5% CO_2_. The remaining raw saliva was stored at −80°C. After incubation, all bacterial growth was harvested into 2 ml STGG and vigorously vortexed until homogenized. Two hundred microliters of culture-enriched saliva was used for DNA extraction, and the remaining samples were stored at −80°C.

For both raw and culture-enriched saliva samples, thawed suspensions were pelleted in a 1.5-ml tube at 20,238 × *g* for 10 min. The pellet was resuspended in 300 μl of lysis buffer with protease (Agowa Mag mini DNA extraction kit; LGC Genomics, Berlin, Germany), 100 μl of sterilized zirconia/silica beads (diameter of 0.1 mm; BioSpec Products, Bartlesville, OK, USA), and 300 μl of phenol (Phenol BioUltra; Sigma-Aldrich, Zwijndrecht, The Netherlands). The sample was mechanically disrupted by bead beating in a TissueLyser LT (Qiagen, Venlo, The Netherlands) twice at 50 Hz for 3 min. After 10 min centrifugation at 9,391 × *g*, the aqueous phase was transferred to a sterile 1.5-ml tube. Binding buffer was added at twice the volume of the aqueous phase plus 10 μl of magnetic beads, after which the sample was incubated in a mixing machine (∼265 rpm) for 30 min at room temperature. The magnetic beads were washed with 200 μl of both wash buffer 1 and wash buffer 2 and eluted with 63 μl of elution buffer according to the manufacturer's instructions. For optimization experiments, DNA from all raw saliva samples was also extracted using QIAamp DNA minikit (Qiagen, Manchester, UK) following manufacturer’s instructions. This method showed the same results as the one described previously in this section.

### Bacterial DNA extraction from nasal fluid pellet.

On the day of the extraction, nasosorption filter strips were thawed for 30 min at room temperature. One hundred microliters of assay diluent was added to the filter and centrifuged at 1,503 × *g* for 10 min. After centrifugation, the eluted liquid was moved to a clean Eppendorf tube and centrifuged at 16,000 × *g* for 10 min at 4°C. The supernatant was removed and used for cytokine analysis, whereas the pellet was used for DNA extraction. Bacterial genomic DNA was extracted from the nasal fluid pellets using the same method as described above (saliva samples).

### Quantification of pneumococcal DNA by qPCR in saliva and nasal fluid pellet samples.

Colonization density was determined by 6A/B-specific qPCR targeting the CpsA gene using the Mx3005P system (Agilent Technologies, Cheadle, UK). The primers and probe sequences were as follows: forward primer, 5′-AAGTTTGCACTAGAGTATGGGAAGGT-3′; reverse primer, 5′-ACATTATGTCCATGTCTTCGATACAAG-3′; and probe, 5′-(FAM)-TGTTCTGCCCTGAGCAACTGG-(BHQ1)-3′ (22). The 25-μl PCR mix consisted of 12.5 μl 1× TaqMan Universal PCR master mix (Life Technologies Ltd, Paisley, UK), 0.1 μl 100 μM each primer, 0.05 μl 100 μM probe, 9.75 μl molecular graded water (Fisher Scientific, Loughborough, UK), and 2.5 μl of the extracted DNA. Thermal cycling conditions were as follows: 10 min at 95°C and 40 cycles of 15 s at 95°C and 1 min at 60°C. A negative DNA extraction control (parallel extraction from sample buffer only), a qPCR-negative control (master mix only), and three extractions of each sample were amplified. A standard curve of a 10-fold dilution series of genomic DNA extracted from S. pneumoniae 6B was used. The genomic DNA was extracted with the QIAamp DNA minikit (Qiagen, Manchester, UK) and quantified with a spectrophotometer (NanoDrop ND-1000; Thermo Fisher Scientific, Landsmeer, The Netherlands). To convert the weight of pneumococcal DNA to number of S. pneumoniae DNA copies, the weight of one genome copy of TIGR4 was used to calculate the genome length in base pairs times the weight of a DNA base pair (650 Da). Samples were considered positive if two or all triplicates yielded a *C_T_* value of <40 cycles. Multiple experiment analysis was performed, and cross experiment threshold was calculated by using interrun calibrators.

### Human myeloperoxidase ELISA.

Levels of myeloperoxidase were determined using the human myeloperoxidase DuoSet ELISA kit (R&D Systems, Abingdon, UK). Ninety-six-well enzyme-linked immunosorbent assay (ELISA) plates were coated with 4 μg/ml capture antibody in phosphate-buffered saline (PBS) at room temperature (RT) overnight. Plates were washed 3 times with PBS (Sigma-Aldrich Co. Ltd, Irvine, UK) containing 0.05% Tween 20 (Sigma-Aldrich Co. Ltd, Irvine, UK) between each step. Wells were blocked with 1% bovine serum albumin (BSA) in PBS for 1 h at RT. Samples and standards were diluted in 1% BSA-PBS in the precoated plates and incubated at RT for 2 h. Detection was performed by incubating plates with detection antibody at 50 ng/ml for 2 h at RT, followed by 20 min incubation with streptavidin-horseradish peroxidase (HRP) (1:200) (Fisher Scientific, Loughborough, UK) at RT. Signal was developed using TMB-Turbo substrate (Fisher Scientific, Loughborough, UK) for 20 min and stopped by adding 2 N H_2_SO4 in a 1:1 ratio. Optical density reading was performed at 450 nm and corrected for optical imperfection (540 nm). All samples were run in duplicate. Results are expressed as micrograms per milliliter and calculated using an MPO standard curve.

### Agglutination assay by flow cytometry.

S. pneumoniae 6B cells were grown to mid-log phase and stored at −80°C in glycerol until use as described previously (23). For agglutination assays with human nasal wash samples, cells were thawed and washed with PBS, and 4 × 10^5^ CFU bacteria in 2 μl saline was incubated with 48 μl of concentrated nasal wash supernatant (1 ml of nasal wash concentrated to 50 μl using vacuum concentrator RVC 2-18) and dialyzed overnight in PBS using Slide-A-Lyzer dialysis units (Thermo Fisher). Antiserum to group 6 (Statens Serum Institut; Neufeld antisera to group 6) was used as a positive control and anti-Hep-A purified human IgG was used as a negative control (using Sepharose and pooled sera from HepA-vaccinated volunteers). Samples were vortexed lightly and incubated for 1.5 h at 37°C and 5% CO_2_.

Cells were fixed with paraformaldehyde (PFA) and analyzed on a BD LSR II flow cytometer (BD Biosciences, San Jose, CA, USA). Bacterial population was gated in the forward scatter (FSC) and sideward scatter (SSC) dot plot referring to cell size and granularity. Photomultiplier tubes (PMT) voltages and threshold were gated on negative control bacteria. A total of 30,000 events for each sample were measured by triplicate using FacsDiva software 6.1 (BD Biosciences, San Jose, CA, USA). Agglutination was quantified by calculating the proportion of the bacterial population with altered FSC and SSC, and values were expressed as percentage of agglutination as previously described (37). All samples were analyzed in duplicate, and 30,000 events were acquired using FacsDiva software 6.1 (BD Biosciences, San Jose, CA, USA). Analysis was performed using FlowJo software version 10.0 (Tree Star Inc., San Carlos, CA, USA).

### Nasal cells processing and flow cytometry.

Cells were dislodged from the curette by repeated pipetting with PBS+ as described previously (19, 27). Cells were spun down at 440 × *g* for 5 min and resuspended in PBS++ containing LIVE/DEAD fixable aqua dead cell stain (Thermo Fisher). After 15 min incubation on ice, an antibody cocktail, which included EpCam-PE, HLADR-PECy7, CD66b-FITC, CD19-BV650 (all BioLegend), CD3-APCCy7, CD14-PercpCy5.5 (BD Biosciences), and CD45-PACOrange (Thermo Fisher), was added to the cells. Following a further 15-min incubation on ice, cells were filtered over a 70-μm filter (Thermo Fisher). Cells were spun down (440 × *g* for 5 min), resuspended in PBS containing 0.5% heat-inactivated fetal bovine serum and 5 mM EDTA (Invitrogen), and acquired on a flow cytometer (LSR II; BD). All cells per tube were acquired, and samples with less than 500 immune cells or 250 epithelial cells were excluded from further analysis (9/40 samples; 22.5%). The numbers of acquired cells per population were used as counts. Flow cytometry data were analyzed using FlowJo version 10 (Tree Star Inc., San Carlos, CA, USA).

### Cytokine analysis.

Nasal washes were centrifuged at 1,503 × *g* for 10 min, and the extracted supernatant was stored at −80°C until use. Human MPO was measured according to the manufacturer’s instructions. The human magnetic 30-plex cytokine kit (Thermo Fisher) was used to detect 30 cytokines simultaneously on an LX200 with xPonent 3.1 software (Luminex) following manufacturer’s instructions from centrifuged nasal lining fluid. Analytes with a coefficient of variation (CV) of >50% were excluded from further analyses. One Luminex plate did not pass quality control, and samples with remaining volume were reanalyzed.

### Heatmap generation and total cytokine score.

Heat map representations were generated using R. Fold change concentrations to baseline were calculated for each individual and log_2_ transformed. An average fold change for each group for each time point was then calculated. A total cytokine score was calculated as previously described (30). In brief, Z-scores of fold changes for all upregulated cytokines for a given individual were calculated by replacing downregulated cytokines with a value of 0 and then summing the Z-scores per sample.

### Quantification and statistical analysis.

Statistical analysis was performed using GraphPad Prism version 5.0 (CA, USA) and R software. Data was log transformed where appropriate. To distinguish between parametric and nonparametric data, a Kolmogorov-Smirnoff test was performed. If two parametric groups were compared, a two-tailed *t* test was used for unpaired and paired groups. If two nonparametric groups were compared, a Mann-Whitney or Wilcoxon test was used for unpaired and paired groups, respectively. If multiple unmatched groups were compared, a one-way analysis of variance (ANOVA) (followed by a Tukey’s posttest) or Kruskal-Wallis test (followed by a Dunn’s posttest) was used for parametric or nonparametric groups, respectively. For Luminex data, a Benjamini-Hochberg correction was used to account for testing of 30 cytokines simultaneously. To quantify association between groups, a Pearson or Spearman correlation test was used for parametric or nonparametric groups, respectively. Differences were considered significant if *P* was <0.05.
