# Supplementary material for: Experimental Human Challenge Defines Distinct Pneumococcal Kinetic Profiles and Mucosal Responses between Colonized and Non-Colonized Adults
Source: mBio. 2021 Jan 12;12(1):e02020-20. doi: 10.1128/mBio.02020-20 (PMC7844534; doi:10.1128/mBio.02020-20)
Supplement: TABLE S1 [file mBio.02020-20-st001.docx]

**Table S1. Volunteers’ demographic data.** Sixty-three volunteers aged 18-49 years: 41 culture-negatives and 22 culture-positives

|  | **Culture-negative (n=41) *** | **Culture-positive (n=22) *** |
| --- | --- | --- |
| **Mean age in years (SD)** | 22 (3.1) | 23 (6.6) |
| **No. Female/Male** | 29/12 | 16/6 |

*One culture-negative and one culture-positive volunteers did not store samples at the correct temperature and were excluded from further analyses.
