# Supplementary material for: Experimental Human Challenge Defines Distinct Pneumococcal Kinetic Profiles and Mucosal Responses between Colonized and Non-Colonized Adults
Source: mBio. 2021 Jan 12;12(1):e02020-20. doi: 10.1128/mBio.02020-20 (PMC7844534; doi:10.1128/mBio.02020-20)
Supplement: FIG S1 [file mBio.02020-20-sf001.docx]

**
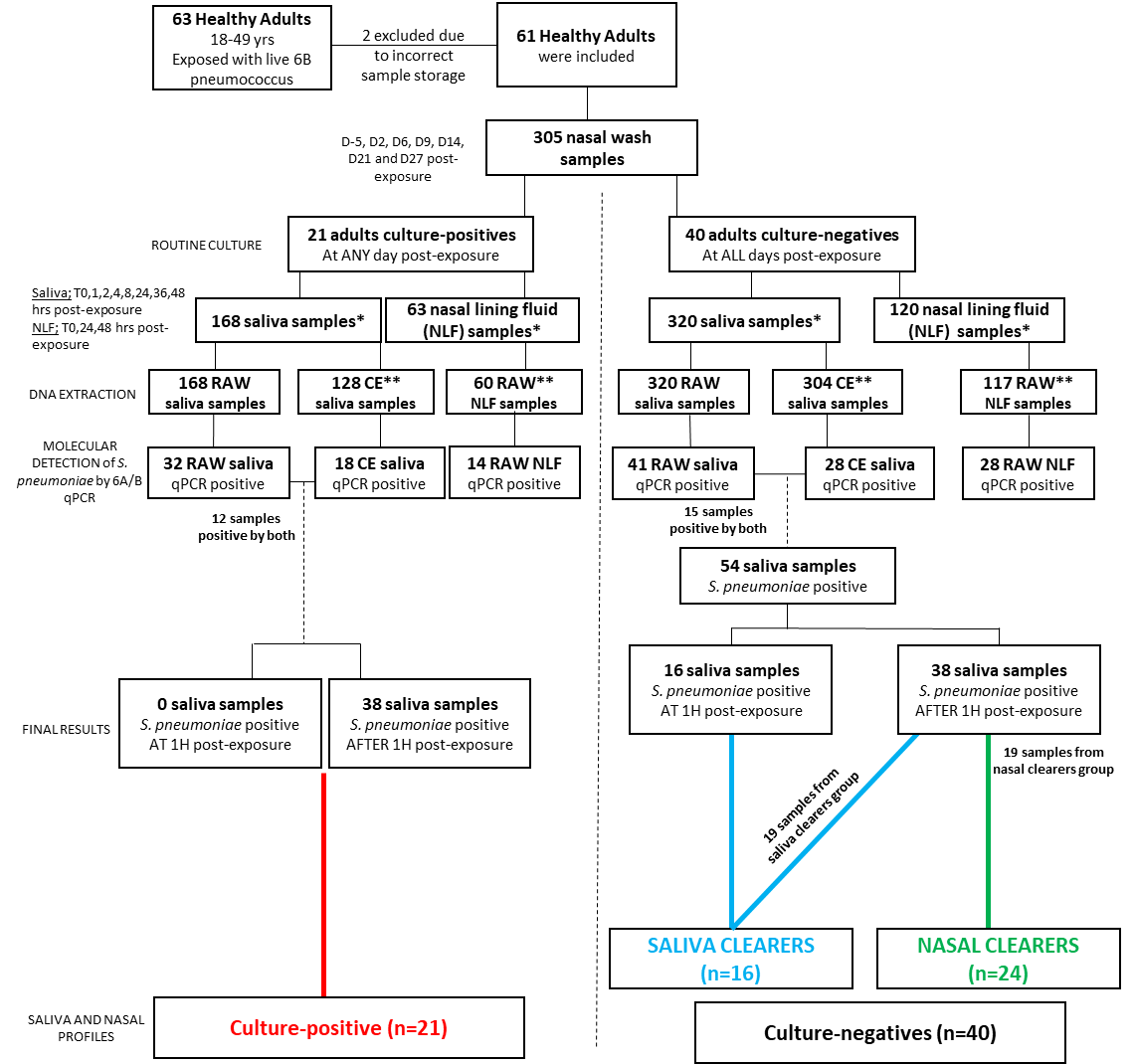
**

**Fig. S1 Flow chart depicting the samples processing for pneumococcal detection in the study.** Sixty-three healthy young adults age 18-49 years were recruited. Two volunteers were excluded from the initial cohort due to incorrect sample storage as detected by temperature monitoring, thus 61 volunteers were used in this analysis. Colonization status was defined by classical microbiology culture (blood agar plates with gentamycin) of *S. pneumoniae* serotype 6B in nasal wash samples collected before (day -5) and at days 2, 6, 9,14, 21 and 27 post-exposure. Twenty-one volunteers were classified culture-positives as they had at least one positive sample at any time point during the study, whereas forty volunteers had negative samples at all time points and classified as culture-negatives. Saliva and NLF samples were obtained before exposure (Time=0 hours, baseline, day of exposure) from all volunteers. Volunteers self-collected their own saliva at 1, 2, 4, 8, 24, 36, and 48 hours, and NLF (by nasosorption strip) at 24 and 48 hours post-exposure. Pneumococcal presence was detected in both saliva and NLF samples by extracting pneumococcal genomic DNA from raw material. In addition, for saliva samples, DNA was extracted from culture-enriched (CE; blood agar plate with gentamicin) saliva samples. *A subset of 33 volunteers self-collected in addition NLF samples at 4 and 8 hours (66 samples) post-exposure; these numbers are not included in this diagram. **Samples from 7 volunteers (five culture-positives, 40 samples and two culture-negatives, 16 samples) were used for optimization experiments and were not included for culture-enriched extraction. From two of these seven volunteers (one culture-positive, 3 samples and one-culture negative, 3 samples) no bacterial DNA was extracted from NLF).
