# Supplementary material for: Experimental Human Challenge Defines Distinct Pneumococcal Kinetic Profiles and Mucosal Responses between Colonized and Non-Colonized Adults
Source: mBio. 2021 Jan 12;12(1):e02020-20. doi: 10.1128/mBio.02020-20 (PMC7844534; doi:10.1128/mBio.02020-20)
Supplement: FIG S2 [file mBio.02020-20-sf002.docx]

**
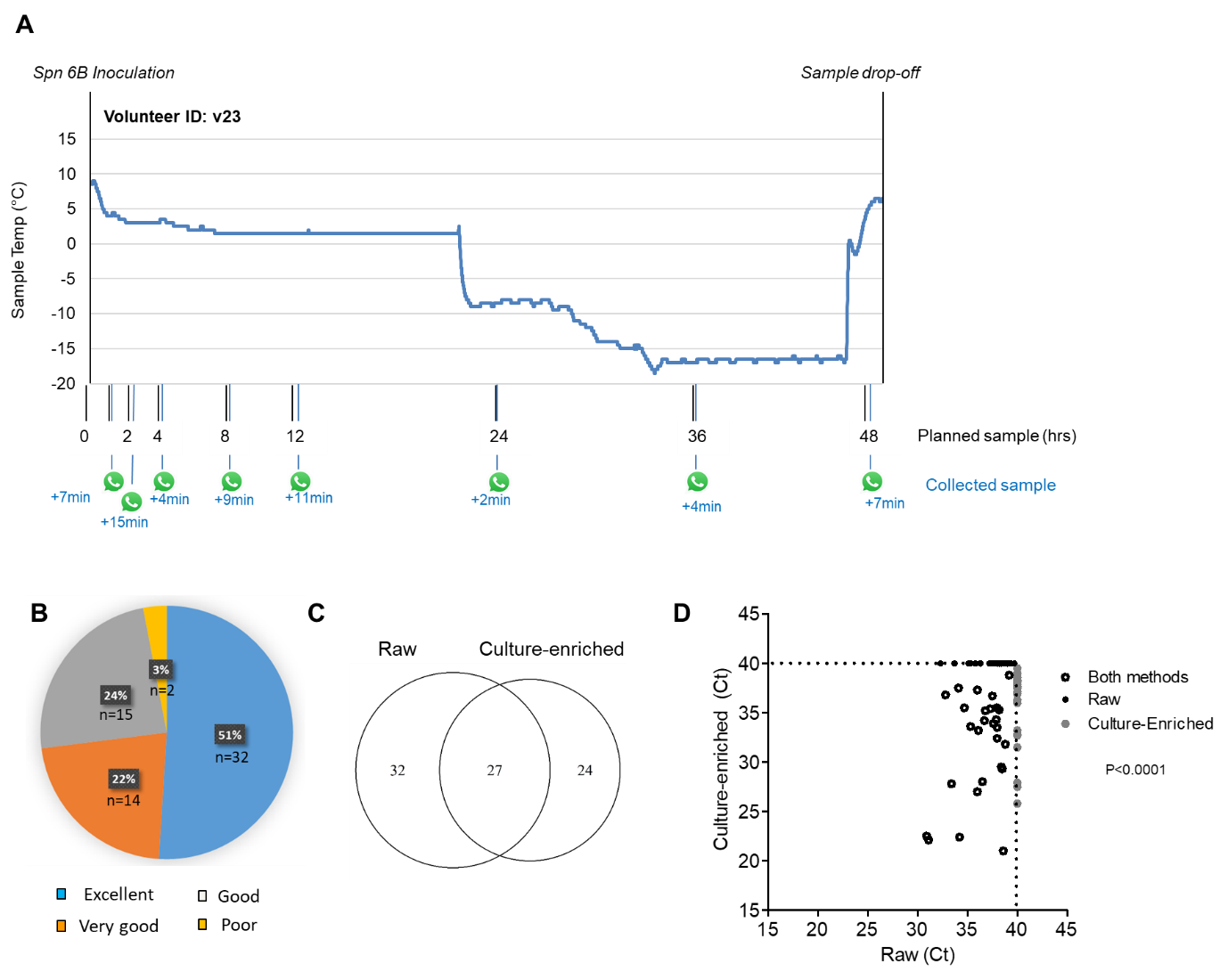
**

**Fig. S2 Home sampling of saliva and nasal lining fluid for Spn detection.** **(A)** Compliance monitoring for one example volunteer (v23). Temperature loggers were used to measure sample temperature and recordings were taken every 20 seconds to ensure sample stability. Volunteers sent pictures of collected samples directly after sample collection to demonstrate they were taken at the correct time. Planned times relative to experimental Spn inoculation are shown and compared with the times of pictures for one representative volunteer. **(B)** Pie chart of compliance rates for all 63 volunteers. The number of corrected stored samples was counted per volunteer, expressed as percentage (%) of the total number of samples and used to categorize compliance status. Compliance status was then expressed as % compliance to the total number of volunteers. Excellent: 100% samples stored at fridge/freezer, very good: 80-90% samples stored at fridge/freezer, good: 70-80% samples stored at fridge/freezer and poor: ≤ 50% samples stored at fridge/freezer from time of collection. **(C)** Venn diagram of Spn detection using qPCR in culture-enriched saliva versus raw extracted saliva. Saliva samples from 7 volunteers (v4, v5, v7, v8, v9, v21, v38 with 17 positive Spn timepoints) were excluded as no culture-enrichment step was performed for these volunteers, DNA from all material was already extracted before they were plated. **(D)** Comparison of Spn6B density from raw extracted and culture-enriched saliva samples. Open circles are samples detected by both methods (n=27). Spn density in samples detected by culture-enriched was statistically significantly higher than those detected by raw DNA extraction (****P<0.0001, paired *t* test). Black circles are samples detected only by raw (n=32) and grey circles are samples detected only by culture-enriched DNA extraction (n=24).
