# Supplementary material for: Experimental Human Challenge Defines Distinct Pneumococcal Kinetic Profiles and Mucosal Responses between Colonized and Non-Colonized Adults
Source: mBio. 2021 Jan 12;12(1):e02020-20. doi: 10.1128/mBio.02020-20 (PMC7844534; doi:10.1128/mBio.02020-20)
Supplement: FIG S3 [file mBio.02020-20-sf003.docx]

**
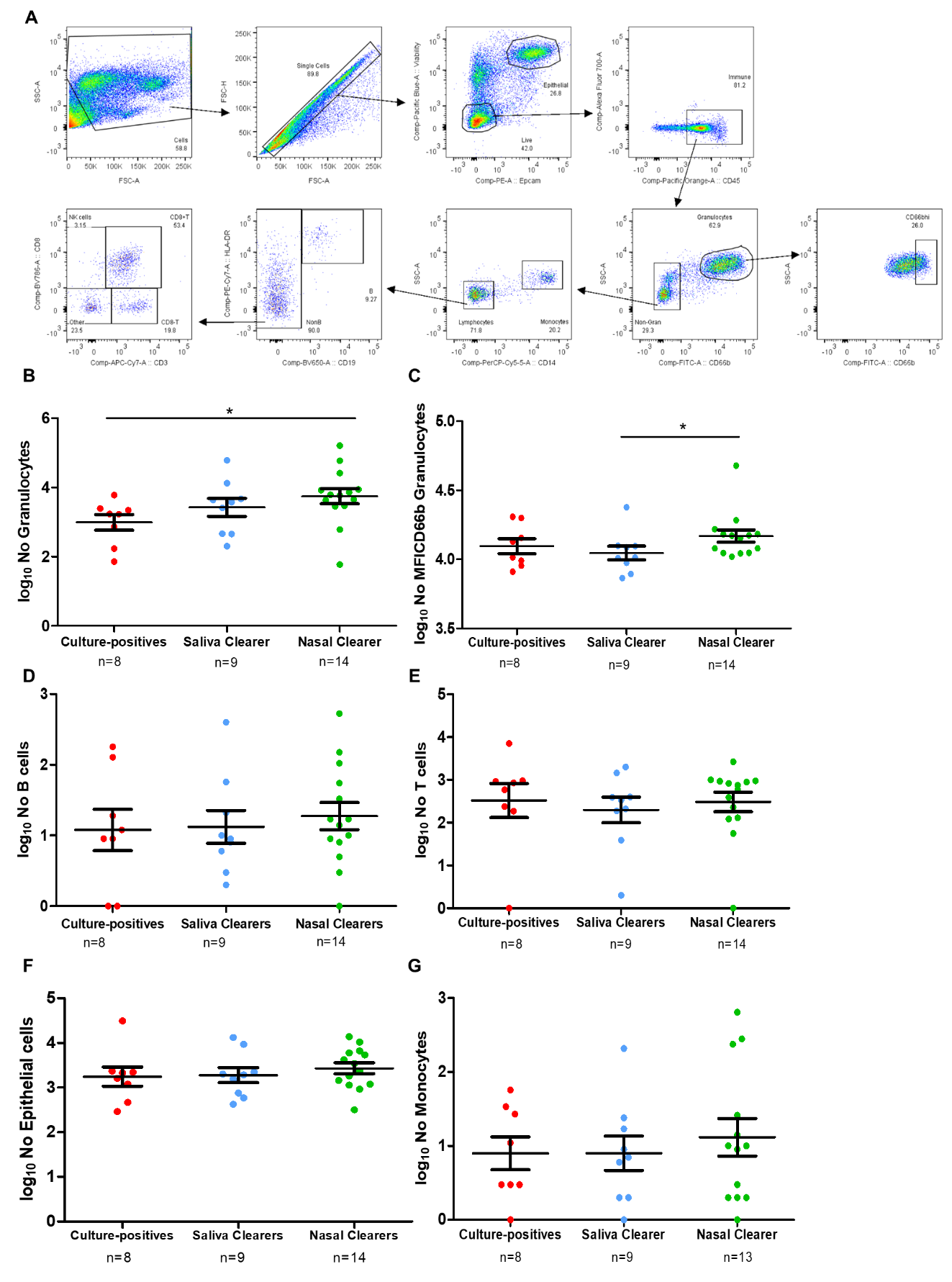
**

**Fig. S3 Nasal immune and epithelial cells measured from nasal curettes at baseline. (A)** Gating strategy for one representative volunteer. **(B)** Numbers of granulocytes at baseline.  *P=0.038, unpaired *t* test. **(C)** Mean fluorescent intensity (MFI) of CD66b on granulocytes. *P=0.047, Mann-Whitney. **(D)** Numbers of B cells at baseline. **(E)** Numbers of T cells at baseline. **(F)** Numbers of epithelial cells at baseline. **(G)** Numbers of monocytes at baseline.  Each dot represents a volunteer. Data was log transformed after adding 1 to all values to allow transforming 0 values and are represented as mean ± SEM.
