# Supplementary material for: Experimental Human Challenge Defines Distinct Pneumococcal Kinetic Profiles and Mucosal Responses between Colonized and Non-Colonized Adults
Source: mBio. 2021 Jan 12;12(1):e02020-20. doi: 10.1128/mBio.02020-20 (PMC7844534; doi:10.1128/mBio.02020-20)
Supplement: FIG S4 [file mBio.02020-20-sf004.docx]

**
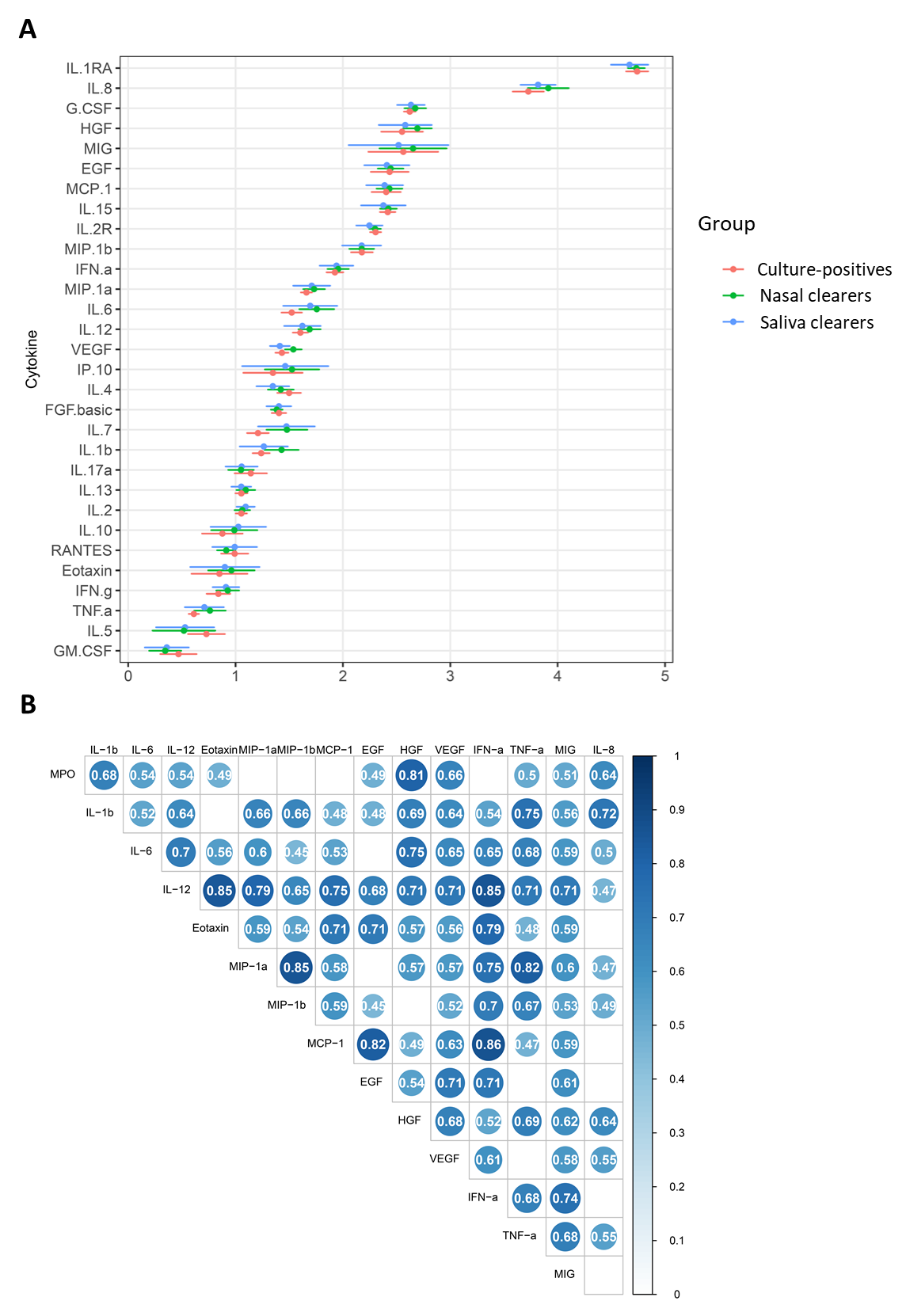
**

**Fig. S4 Nasal cytokine levels prior to challenge. (A)** Nasal cytokines at baseline. Mean and 95% confidence intervals are shown for log-transformed cytokine concentrations (pg/mL) for culture positives (red), nasal clearers (green) and saliva clearers (blue) in nasosorption. **(B)** Correlation matrix showing the association between significantly induced cytokines and MPO levels in nasosorption at 24 hours in saliva clearer and culture-positive groups. Non highly significant correlations (P > 0.001, Spearman test) are left blank and rho values are shown for each cytokine pair. Colour and size reflects strength of a correlation.
